# Galectin-9 expression predicts poor prognosis in hepatitis B virus-associated hepatocellular carcinoma

**DOI:** 10.18632/aging.203909

**Published:** 2022-02-24

**Authors:** Jianhua Jiao, Dian Jiao, Fa Yang, Jingliang Zhang, Yu Li, Donghui Han, Keying Zhang, Yingmei Wang, Rui Zhang, An-Gang Yang, Anhui Wang, Weihong Wen, Weijun Qin

**Affiliations:** 1 Department of Urology, Xijing Hospital, Fourth Military Medical University, Xi’an 710032, China; 2 Department of Urology, Tangdu Hospital, Fourth Military Medical University, Xi’an 710032, China; 3 Department of Health Services, Health Service Training Base, Fourth Military Medical University, Xi’an 710032, China; 4 State Key Laboratory of Cancer Biology, Department of Pathology, Xijing Hospital and School of Basic Medicine, Fourth Military Medical University, Xi’an 710032, China; 5 State Key Laboratory of Cancer Biology, Department of Immunology, Fourth Military Medical University, Xi’an 710032, China; 6 Department of Epidemiology, School of Military Preventive Medicine, Fourth Military Medical University, Xi'an 710032, China; 7 Institute of Medical Research, Northwestern Polytechnical University, Xi’an 710072, China

**Keywords:** HCC, prognosis, Galectin-9, Kupffer cell

## Abstract

Objectives: The aim of this study was to explore the expression of Galectin-9 in hepatitis B virus (HBV)-associated hepatocellular carcinoma (HCC), evaluate its clinicopathological significance, and investigate whether Galecin-9 expression has prognostic value in HBV-associated HCC.

Methods: Immunohistochemistry staining was performed to examine the expression of Galectin-9 in paraffin-embedded tissues from 140 cases of HBV-associated HCC specimens. The association between Gal-9 expression, clinicopathological features and prognosis was analyzed by Kaplan-Meier method, log-rank test and Cox regression analysis. Dual immunofluorescence (IF) staining was performed to identify the cell types that have positive Gal-9 expression.

Results: Among the 140 cases of HBV-associated HCC, 39 (27.9%) cases showed high Gal-9 expression (score≥6), 21 (15%) cases showed moderate Gal-9 expression (6>score≥3), 33 (23.6%) cases showed weak Gal-9 expression (3>score>0), and 47 (33.6%) cases had no detectable Gal-9 expression (score=0). Positive Gal-9 expression (score>0) was associated with lymph node metastasis (*P*=0.029), Ki-67 proliferation index (*P*=0.009) and poor prognosis. Univariate and multivariate analyses showed that Gal-9 expression could be used as an independent prognostic marker for HBV-associated HCC. Dual IF staining indicated that Gal-9 was mainly expressed in CD68^+^CD163^+^ Kupffer cells (KCs) in HBV-associated HCC.

Conclusions: Gal-9 was specifically expressed in certain HBV-associated HCC. Positive Gal-9 expression was significantly associated with poor prognosis, and Gal-9 could be used as a prognostic marker in HBV-associated HCC. Specific expression of Gal-9 on KCs indicated it may have immunosuppressive function in HBV-associated HCC.

## INTRODUCTION

With more than 900 thousand new cases and 830 thousand deaths in 2020, liver cancer ranked as the 6^th^ most commonly diagnosed malignancies and third cancer-related cause of death worldwide [1]. As the main pathological type of liver cancer, hepatocellular carcinoma (HCC) accounts for 75-85% of all cases and chronic hepatitis B virus (HBV) infection is one major risk for HCC [2]. Across the world, more than 250 million people are HBV carriers, and new-born children from HBV-infected mothers also become chronic carriers, they are destined to develop HCC [3]. Thus, it is essential to identify prognostic biomarkers in HBV-associated HCC.

Galectins belong to the lectin family that bind β-galactoside through highly conserved carbohydrate recognition domains (CRDs). Galectin-9 (Gal-9) is the subgroup of tandem repeat-type galectins, since it contains 2 conserved CRDs [4, 5]. So far, Gal-9 has been identified as the ligand of several receptors, such as T cell immunoglobulin- and mucin-domain-containing molecule (Tim-3), CD44 and Dectin 1. Gal-9 could inhibit immune response through different mechanisms [6]. For example, Gal-9 could induce Th1 cell death through Tim-3, thus inhibiting Th1 immunity [7]. Gal-9 could increase iTreg cell stability and function through the interaction with CD44 [8]. And Gal-9 could induce macrophage mediated adaptive immune suppression through its binding to Dectin 1 [9].

Recent years, Gal-9 has been found to have prognostic value in certain cancer types. For example, in pancreatic cancer, Sun et al. reported that higher Gal-9 expression was associated with better disease-free survival (DFS) and overall survival (OS) [10]. For breast cancer, Irie et al. found that patients with Gal-9^+^ tumors had a more favorable DFS than patients with Gal-9^-^ tumors, and lower Gal-9 expression could predict a relatively higher risk of metastasis [11]. In ovarian cancer, Schulz. et al. found that moderate expression of Gal-9 could predict poorer outcome than Gal-9 negative cases, however, it seemed that patients with strong Gal-9 expression predicted best outcome [12]. In lung adenocarcinoma, patients with high Gal-9 expression had shorter survival time than patients with low or negative Gal-9 expression [13]. In sum, the prognostic value of Gal-9 is inconsistent in different cancer types.

In HCC, several studies have shown that Gal-9 expression also have prognostic value. For example, in one study, Li et al. reported that Gal-9 was highly expressed in Kupffer cells (KCs), which were colocalized with Tim-3 positive T cells in HBV-associated HCC patients. They also found that positive Tim-3 expression predicted poor survival in HBV-associated HCC patients, and blocking the interaction between Tim-3 and Gal-9 could increase the function of Tim-3 positive T cells [14]. However, they didn’t evaluate the prognostic value of Gal-9 in their study. Several other studies showed different results. Sideras et al. found that 79% HCC patients had Gal-9 expression, while Gal-9 mainly expressed in tumor cells, they also found that low Gal-9 and PD-L1 expression level and low number of CD8^+^ tumor-infiltrating lymphocyte (TILs) were associated with reduced outcome in HCC [15]. Besides, they also found that high level of circulating Gal-9 could also predict better survival in HCC. Furthermore, combined analysis of intra-tumoral Gal-9 expression and its circulating level could more confidentially predict survival [16]. Zhang et al. found that Gal-9 expression could be detected in 56.5% of HCC patients, Gal-9 was mainly expressed in tumor cells and its expression was closely correlated with histopathological grade, vascular invasion, lymph node metastasis and intrahepatic metastasis. They also found that patients with Gal-9^+^ tumors had longer survival time than those with Gal-9^-^ tumors [17]. According to these studies, the cell type that express Gal-9 was not consistent, and the prognostic value of Gal-9 seemed also controversial. Thus it is necessary to further evaluate the prognostic value of Gal-9 in HCC.

In current study, we examined the expression of Gal-9 in 140 HBV-associated HCC tumor tissues, evaluated the association between Gal-9 expression and clinicopathological features, and also evaluated its prognostic value in HBV-associated HCC. Besides, we also examined the cell types that have positive Gal-9 expression.

## MATERIALS AND METHODS

### Patients with HBV-associated HCC

HBV-associated HCC tumor tissues were collected from 140 patients that received surgery at Xijing Hospital (Xi’an, China) from 2008 to 2013. Tumor specimens were formalin-fixed, paraffin-embedded and stored at the Department of Pathology of Xijing Hospital. After the surgery, patients were followed up with an average period of 51 months (1-116 months). And detailed pathological diagnosis was later on confirmed by two experienced pathologists that are blinded to the study design. The diagnostic standard was based on the seventh edition of AJCC (American Joint Committee on Cancer) staging manual. All of the clinical information was collected from electronical record in Xijing Hospital.

### Immunohistochemistry (IHC) staining

The formalin-fixed tissue samples were routinely processed by IHC staining to examine the expression of Gal-9 with a Gal-9 specific antibody (#54330, Cell Signaling Technology; 1:400), as we previously reported [18]. Detailed protocol of IHC staining was provided in Supplementary Materials.

### Immunohistochemistry evaluation

For the evaluation of Gal-9 expression, first, we observed the slides under low magnification (×100) to find representative Gal-staining area, and then examined the expression of Gal-9 under high magnification (×400). The expression of Gal-9 was carefully assessed in a semiquantitative manner. Details regarding IHC evaluation are provided in Supplementary Materials.

### Immunofluorescent (IF) staining

Slides were applied to IF staining with a Gal-9 specific antibody (#54330, Cell Signaling Technology; 1:400), CD68 antibody (GB13063-1, Wuhan Servicebio Technology, 1:2000), Glypican 3 (GPC3) antibody (ab95363, Abcam, 1:100), CD163 antibody (Ab156769, Abcam, 1:150) or CD206 antibody (GB13438, Wuhan Servicebio Technology, 1:2000), as we previously reported [19]. Detailed procedures for IF staining are provided in Supplementary Materials.

### Evaluation of IF staining

Quantitation of the colocalization between Gal-9 and CD68, GPC3, CD163, CD206 was analyzed by Coloc 2 of Fiji (ImageJ, 1.52i, National Institutes of Health, USA). Detailed procedures about IF staining evaluation are provided in Supplementary Materials.

### Statistical analysis

We performed statistical analysis using SPSS 23.0 statistic software (version 23.0, IBM, Armonk, NY, USA). Descriptive statistics, such as mean, standard deviation, and absolute and relative frequencies, were calculated to define the basic characterizations of the study cohort. Chi-squared test was performed to evaluate the association between the expression of Gal-9 and patients’ clinicopathological features. We generated survival curve by Kaplan-Meier method and compared them by log-rank test. Then, the hazard ratios with their 95% confidence intervals (CI) were evaluated by Cox proportional hazards models. *P* < 0.05 was regarded as statistically significant.

### Ethics approval

This study was approved by the ethics committee of Xijing Hospital, Fourth Military Medical University.

### Availability of data and material

Data and material used to support the findings of this study were provided in the article.

## RESULTS

### Expression of Gal-9 in HBV-associated HCC tissues

**Figure 1:**
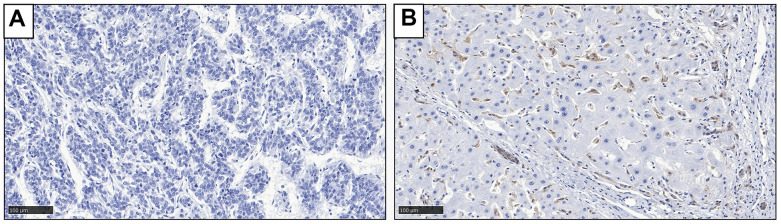
**Representative IHC staining result to show the expression of Gal-9 in HCC tissues.** (**A**) Negative Gal-9 expression; (**B**) Positive Gal-9 expression.

Gal-9 expression was examined in 140 HBV-associated HCC samples. Results showed that, among these samples, 39 (27.9%) cases had strong Gal-9 expression (score≥6), 21 (15%) cases had moderate Gal-9 expression (6>score≥3), 33 (23.6%) cases had weak Gal-9 expression (3>score>0), and 47 (33.6%) cases had no detectable Gal-9 expression (score=0). Representative IHC staining results were shown in Figure 1. These results indicate that Gal-9 is specifically expressed in certain patients with HBV-associated HCC.

### Expression of Gal-9 is correlated with some clinicopathological features in HBV-associated HCC

We provided the clinicopathological features of the 140 HBV-associated HCC patients in Table 1. HBV-associated HCC patients were separated into two groups according to the expression level of Gal-9, that is, cases with strong, moderate and weak Gal-9 expression were all designated as positive Gal-9 expression (score>0), while cases with no detectable Gal-9 expression were designated as negative Gal-9 expression (score=0). Then, the association between Gal-9 expression and clinicopathological features was analyzed, and positive Gal-9 expression was found to be correlated with lymph node metastasis (*P*=0.029) and Ki-67 proliferation index (*P*=0.009), while no association was observed between Gal-9 expression and age, gender, tumor differentiation, tumor stage and tumor-node-metastasis (TNM) stage (Table 2). These results demonstrate that Gal-9 expression is closely associated with some clinicopathological features, that is, lymph node metastasis and proliferation index in HBV-associated HCC.

**Table 1:** Clinicopathological features of 140 HBV-associated HCC patients.

**Clinicopathological features**	**No. of patients (%)**
Gender	
Male	103 (73.6)
Female	37 (26.4)
Age, yrs	
Median	56
Range	17-82
Tumor differentiation	
Well	52 (37.1)
Moderately/ Poorly	88 (62.9)
T stage	
T1	77 (55.0)
T2	36 (25.7)
T3	25 (17.9)
T4	2 (1.4)
N stage	
N0	120 (85.7)
N1	20 (14.3)
M stage	
M0	135 (96.4)
M1	5 (3.6)
TNM stage	
I	74 (52.9)
II	28 (20.0)
III	17 (12.2)
IV	21 (15.0)

**Table 2:** Association between Gal-9 expression and clinicopathological features in HBV-associated HCC patients (n=140).

**Clinicopathological features**	**No. of patients**		**Galectin-9 expression**		**χ2**	**P value**
			**Negative**	**Positive**		
Age					0.239	0.625
<60	96	33		63		
≥60	44	17		27		
Gender					0.007	0.932
Male	103	37		66		
Female	37	13		24		
Tumor differentiation						
Well	52	19		33	0.024	0.876
Moderately/poorly	88	31		57		
Tumor stage						
T1-2	114	42		72	0.340	0.560
T3-4	26	8		18		
Lymph node metastasis						
N0	120	47		73	4.361	0.029*
N1	20	3		17		
TNM stage						
I-II	106	42		64	2.904	0.065
III-IV	34	8		26		
Ki-67						
<10%	50	25		25	6.914	0.009*
>10%	90	25		65		

Gal-9, Galectin-9; TNM, tumor-node-metastasis.

*Statistically significant (P<0.05).

### Positive Gal-9 expression predicts poor prognosis in HBV-associated HCC

**Figure 2:**
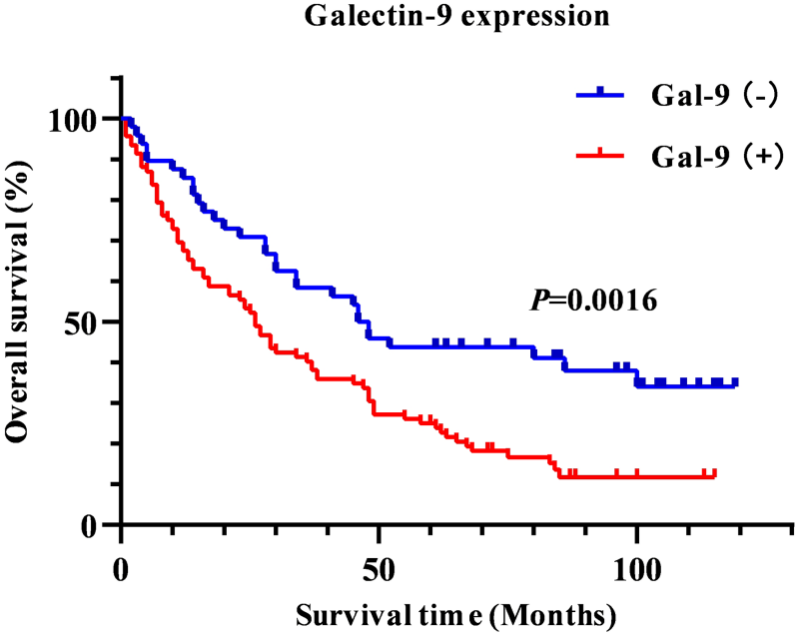
Kaplan-Meier survival curve to show the association between Gal-9 expression and overall survival in HBV-associated HCC patients.

**Figure 3:**
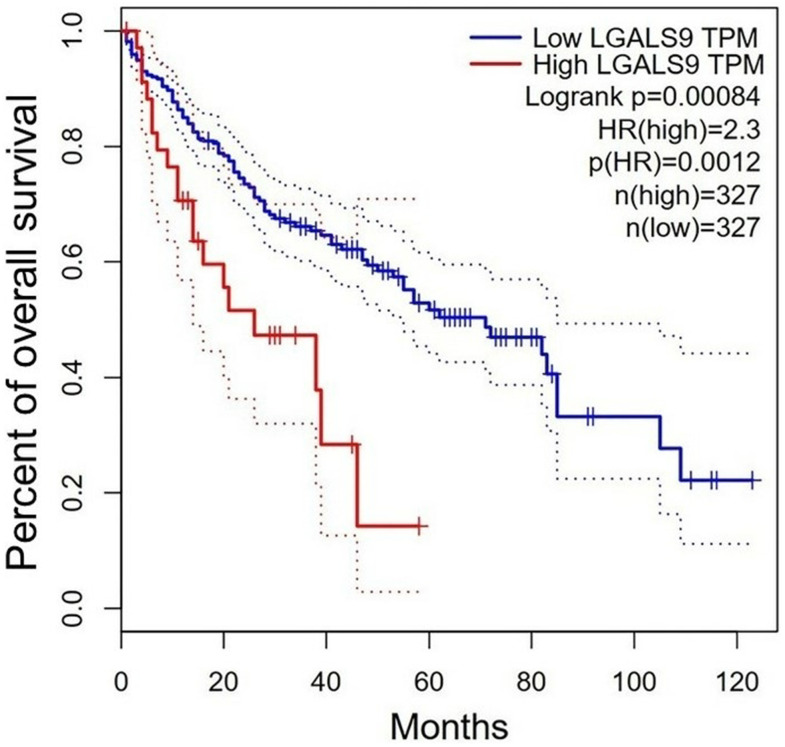
**Survival of HCC patients with different Gal-9 mRNA levels from the GEPIA database.** (http://gepia.cancer-pku.cn/; Cutoff-high (%) = 10; Cutoff-low (%) = 90). HCC patients with high Gal-9 mRNA level have significantly poor survival than those with low Gal-9 mRNA level (*P*=0.0012).

In order to assess the prognostic value of Gal-9 in HBV-associated HCC patients, we investigated the potential association between Gal-9 expression and overall survival (OS). The survival curves of HBV-associated HCC patients with different expression levels of Gal-9 were analyzed by Kaplan-Meier analysis and log-rank test. And significant difference was found between patients with Gal-9^+^ tumor and patients with Gal-9^-^ tumor (*P*=0.0016, Figure 2). We also analyzed the OS of HCC patients with different mRNA levels of Gal-9 in GEPIA database. Results showed that HCC patients with higher Gal-9 mRNA level tended to have poorer OS than patients with lower Gal-9 mRNA level (*P*=0.0012, Figure 3). These results were consistent with our IHC staining results. Then, by using Cox regression analysis, we analyzed the prognostic factors for OS. Results of univariate analysis indicated that Gal-9 expression (*P* =0.002), tumor stage (*P* =0.003), lymph node metastasis (*P* <0.001), distant metastasis (*P* =0.040) and TNM stage (*P* <0.001) were closely associated with the prognosis of HBV-associated HCC patients. In multivariate analysis, results showed that Gal-9 expression (*P* =0.004), tumor stage (*P* =0.015) and lymph node metastasis (*P* <0.001) were associated with OS (Table 3). These results demonstrate that the expression of Gal-9 can be used as an independent prognostic marker in HBV-associated HCC.

**Table 3:** Cox regression analysis of prognostic factors for overall survival in HBV-associated HCC patients (n=140).

	**Univariate**				**Multivariate**		
	**HR**	**95% CI**	**P value**		**HR**	**95% Cl**	**P value**
Gal-9 expression (positive vs. negative)	1.918	1.265-2.908	0.002*		1.877	1.179-2.744	0.004*
Age (≥60 yr vs. <60 yr)	0.879	0.586-1.320	0.535		——	——	——
Gender (female vs. male)	1.186	0.771-1.826	0.437		——	——	——
Tumor stage (T3-4 vs. T1-2)	2.015	1.267-3.206	0.003*		2.811	1.209-5.811	0.015*
Lymph node metastasis (N1 vs. N0)	4.578	2.735-7.662	<0.001*		7.281	3.175-20.742	<0.001*
Distant metastasis (M1 vs. M0)	2.572	1.042-6.344	0.040*		0.545	0.192-1.553	0.256
TNM stage (III-IV vs. I-II)	2.401	1.576-3.657	<0.001*		0.475	0.175-1.290	0.144
Tumor differentiation (poorly vs. moderately/well)	1.235	0.838-1.821	0.286		——	——	——
Ki-67 (≥10% vs. <10%)	1.356	0.911-2.017	0.133		——	——	——

Abbreviations: CI, confidence interval; HR, hazard ratio; TNM, tumor-node-metastasis.

*Statistically significant (P<0.05).

### Gal-9 mainly expresses in CD68^+^CD163^+^ KCs in HBV-associated HCC

**Figure 4:**
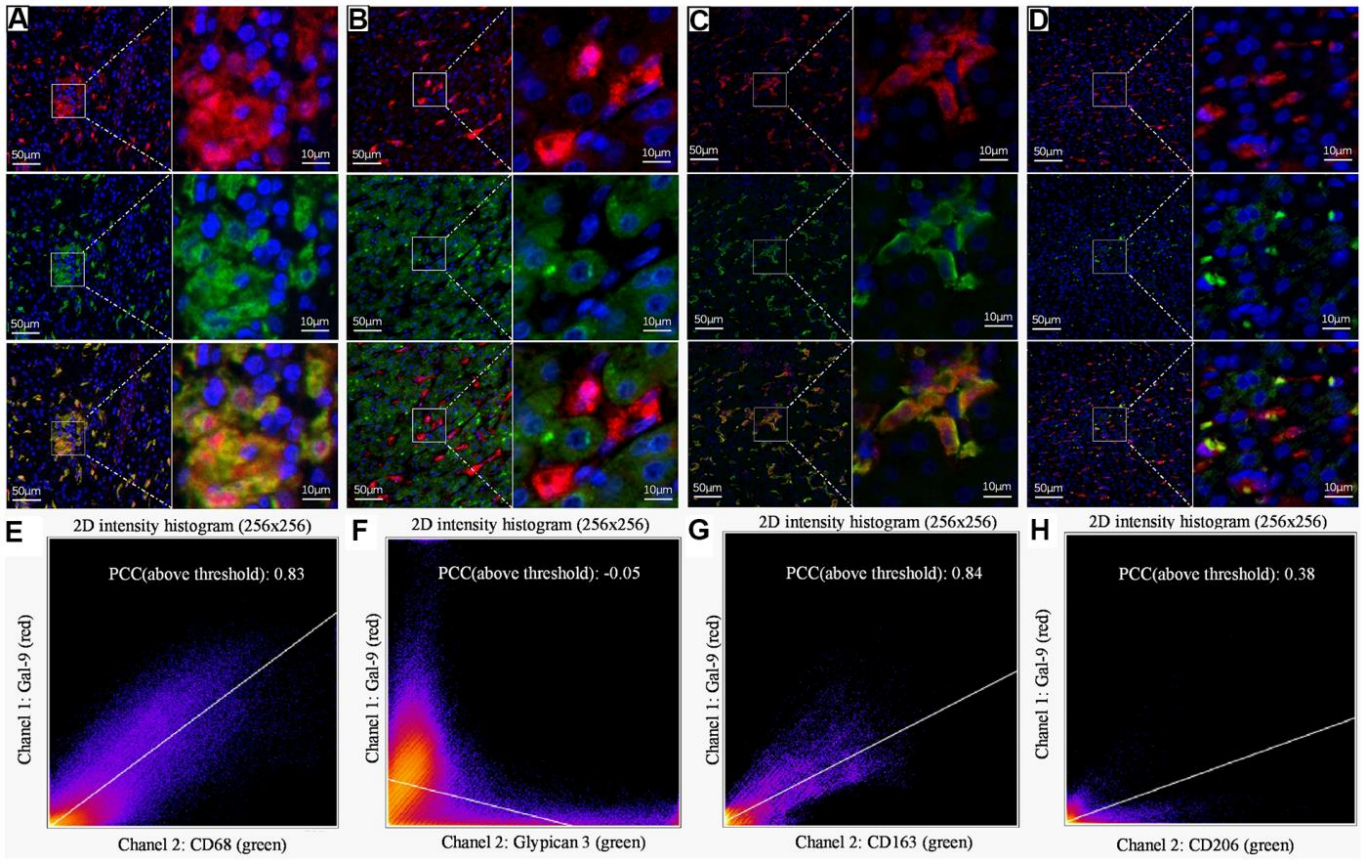
**Representative dual IF staining result to show the colocalization of Gal-9 with different cell markers.** Dual-IF staining of Gal-9 and CD68 (**A**), Gal-9 and GPC3 (**B**), Gal-9 and CD163 (**C**), Gal-9 and CD206 (**D**). (blue: nuclei; red: Gal-9; green: CD68/GPC3/CD163/CD206; yellow: merge). Quantitation of the colocalization of Gal-9 and CD68/GPC3/CD163/CD206 is shown in (**E**–**H**). PCCs (above threshold) of Gal-9 and CD68/GPC3/CD163/CD206 were 0.83, -0.05, 0.84 and 0.38, respectively. Results showed that Gal-9 was mostly expressed on CD68^+^CD163^+^ KCs, but not HCC tumor cells or M2 macrophages. Scale bar, 50μm (left column) and 10μm (right column).

To further evaluate the potential function of Gal-9 in HBV-associated HCC, we analyzed the cell types that had Gal-9 expression. We did dual IF staining using antibodies for Gal-9 and GPC3 (a marker for HCC cells) or CD68 (a marker for macrophages). Result showed that, Gal-9 was mainly colocalized with CD68, indicating that most of the Gal-9 positive cells were macrophages (Figure 4A). Very few colocalization of Gal-9 and GPC3 was observed in all the specimens we examined, indicating that most tumor cells didn’t have Gal-9 expression (Figure 4B). Then we analyzed whether Gal-9 mainly express in KCs or M2 macrophages by dual IF staining using antibodies for Gal-9 and CD163 (a marker for KC activation) or CD206 (a marker for M2 macrophages). Results showed that Gal-9 was mainly colocalized with CD163, but not CD206, indicating that Gal-9 mainly expressed on KCs but not M2 macrophages (Figure 4C, 4D). The quantitation of colocalization of Gal-9 with different markers were shown in Figure 4E–4H. These results indicate that Gal-9 mainly express in CD68^+^CD163^+^ KCs, while barely express in HCC tumor cells or M2 macrophages.

## DISCUSSION

Gal-9, a S-type lectin that belongs to Galectin family, plays an essential role in the regulation of immune response. Gal-9 was identified as a ligand of Tim-3, and its binding with Tim-3 could induce Th1 cell death, thus inhibiting Th1 immunity [7, 20]. Besides direct interaction with Tim3 on T cells, Gal-9 could promote the expansion of CD11b^+^Ly-6G^+^ cells, which represent the granulocytic myeloid-derived suppressor cells (MDSCs), thus indirectly regulate Th1 immune responses [21].

Besides Tim3, Gal-9 has also been identified to interact with other receptors, such as CD44 and Dectin 1. Wu et al. found that Gal-9 was highly expressed in induced regulatory T cells (iTreg), and Gal-9 could bind with CD44 to activate Smad3, thus increase the stability and function of iTreg cells [8]. Gal-9 could also bind with Dectin 1, resulting in macrophage mediated suppression on adaptive immune response in pancreatic ductal adenocarcinoma (PDA) [9]. Recently, Gal-9 was identified as a program death-1 (PD-1)-binding molecule, and the interaction between Gal-9 and PD-1 could attenuate TIM-3/Gal-9-induced cell death, thus regulate the exhaustion of T cells and the efficacy of immunotherapy [22].

Recent years, Gal-9 were found to be specifically expressed in various tumor cells and play a critical role in antitumor immunity. For instance, in gastrointestinal stromal tumors (GISTs), Komita et al. found that the direct interaction between Gal-9 positive tumor cells and Tim-3 positive NK cells may be involved in the suppression of antitumor immunity [23]. In colon cancer, Wang et al. found that Gal-9 could stimulate NK cell migration, thus promote immune surveillance [24]. In glioma, Yuan et al. found that Gal-9 was highly correlated with immune checkpoint molecules and M2 macrophages, indicating a suppressive immune response [25]. In nasopharyngeal carcinoma (NPC), Klibi et al. found that blockade of Galectin-9/Tim-3 interaction could alleviate the Th1-suppressive effect, thus improve antitumoral T-cell responses [26].

Besides tumor cells, Gal-9 has also been found to be specifically expressed in other cell types in different tumors. For example, in early-stage small cell lung cancer (SCLC), Chen et al. demonstrated that Gal-9 was mainly expressed in TILs, and Gal-9 expression was markedly correlated with immune infiltration in the tumor microenvironment. They also found that high expression level of Gal-9 on TILs predicted better recurrence-free survival (RFS) in stage I–III SCLC [27]. In muscle-invasive bladder cancer, Qi et al. found that Gal-9 mainly expressed on tumor-associated macrophages (TAMs), which predicted poor OS and RFS [28]. In osteosarcoma, Gal-9 was found to be expressed in regulatory T cells (Tregs), and its interaction with Tim3^+^ T cells resulted in the suppression in Th1 responses, thus inhibit immune response [29]. In metastatic differentiated thyroid cancer (mDTC), Severson et al. found that Gal-9 was expressed in tumor-involved lymph nodes (TILNs), and Gal-9 may contribute to the dysfunction of PD-1^+^Tim3^+^CD8^+^ T cells, thus promote disease progression [30]. Recently, Isobel et al. showed that expression of Gal-9 was upregulated in both CD8^+^ and CD4^+^T cells in the TILs and peripheral blood in patients with virus-associated solid tumors (VASTs), and Gal-9 expression was also related to the impaired function of T cells [31]. Since Gal-9 expression was indicated to be associated with the formation of suppressive immune microenvironment, Gal-9 was considered as a potential ideal target for cancer therapy [5, 32, 33].

In HCC, the cell types that express Gal-9 is still controversial, and the prognostic value of Gal-9 has not been well studied. In Li’s study, Gal-9 was found to be highly expressed in KCs, while in two other studies, Gal-9 was found to be mainly expressed in tumor cells. In addition, they also got different conclusions in the prognostic value of Gal-9 in HCC. In Li’s study, although they didn’t directly evaluate the prognostic role of Gal-9, they did find that positive Tim-3 expression could predict reduced survival in patients with HBV-associated HCC. Since they found that when the interaction between Tim-3 and Gal-9 was blocked, the function of Tim-3^+^ T cells could increase, indicating that positive expression of Gal-9 in KCs may predict poor survival. While in other three studies, Sideras et al. and Zhang et al. found that patients with Gal-9^+^ tumors seemed to have longer survival time than patients Gal-9^-^ tumors [14–17]. These discrepancies might come from the different patients they studied, Li et al. specifically studied HBV-associated HCC patients, while the other two groups didn’t specify a subclass of the HCC patients. Because of these discrepancies, more studies are needed to confirm the cell types that express Gal-9 and the prognostic value of Gal-9 in HCC patients.

In our study, we examined Gal-9 expression in 140 HBV-associated HCC patients and found that Gal-9 was specifically expressed in certain patients, and Gal-9 expression was closely correlated with some clinicopathological features, that is, lymph node metastasis (*P* =0.029) and Ki-67 proliferation index (*P* =0.009). We also found that patients with Gal-9^+^ tumors tended to have shorter survival time than patients with Gal-9^-^ tumors. Our Cox regression analysis results showed that Gal-9 could be an independent prognostic marker for HBV-associated HCC. In addition, by using dual IF staining, we confirmed that Gal-9 was mainly expressed in CD68^+^CD163^+^ KCs rather than tumor cells or M2 macrophages, indicating that Gal-9 may be involved in the formation of immune suppressive microenvironment in HBV-associated HCC.

Our data are more consistent with Li’s results, we think it’s because that we all used tissue samples from HBV-associated HCC patients. In Li’s study, they also showed CD68+ KCs had highest Gal-9 expression in HCC by flow cytometry. Yet, in their study, they only evaluated the prognostic value of Tim-3, but not Gal-9 [14]. Here, in this study, we confirmed that positive expression of Gal-9 was significantly associated with poor prognosis, that is, Gal-9 had prognostic value in HBV-associated HCC. Since Li’s study showed that the Tim-3^+^ T cell function can be increased by blocking the interaction between Tim-3 and Gal-9, we proposed that expression of Gal-9 in KCs may mediate a suppressive function on anti-tumor T cell response.

Our data is not consistent with the other two studies, which might be caused by the different group of samples we used. We specifically studied Gal-9 expression in HBV-associated HCC, but the other two studies didn’t specify the subclass of HCC. More studies are still needed to examine the prognostic value of Gal-9 in HCC patients that are not HBV-associated. Besides, although our results demonstrated that KCs specifically expressed Gal-9 in HBV-associated HCC, however, how Gal-9 expression was regulated in KCs is not known. Recently, Selno et al. reported that transforming growth factor beta type 1 (TGF-β)/Smad3 pathway could regulate the expression of Gal-9 in colorectal cancer [34]. And Chen et al. reported that EZH2 could promote Gal-9 expression through miR-22 in HCC cells [35]. Further study was also needed to verify whether Gal-9 expression is also regulated through similar mechanisms in KCs in HBV-associated HCC.

In brief, in this study we confirmed that Gal-9 is specifically expressed in certain HBV-associated HCC patients, and positive Gal-9 expression is closely correlated with some clinicopathological features, that is, lymph node metastasis and proliferation index, and also poor prognosis. Furthermore, Gal-9 is mainly expressed in CD68^+^CD163^+^ KCs, indicating it may promote tumor progression through the induction of immunosuppressive microenvironment. Our results suggested that Gal-9 could be a new marker for prognosis and a potential target for the therapy of HBV-associated HCC.

## Supplementary Material

Supplementary Methods

## References

[1] Sung H, Ferlay J, Siegel RL, Laversanne M, Soerjomataram I, Jemal A, Bray F. Global cancer statistics 2020: GLOBOCAN estimates of incidence and mortality worldwide for 36 cancers in 185 countries. CA Cancer J Clin. 2021; 71:209–49. 10.3322/caac.21660 33538338

[2] Bray F, Ferlay J, Soerjomataram I, Siegel RL, Torre LA, Jemal A. Global cancer statistics 2018: GLOBOCAN estimates of incidence and mortality worldwide for 36 cancers in 185 countries. CA Cancer J Clin. 2018; 68:394–424. 10.3322/caac.21492 30207593

[3] Mani SK, Andrisani O. Hepatitis B Virus-Associated Hepatocellular Carcinoma and Hepatic Cancer Stem Cells. Genes (Basel). 2018; 9:E137. 10.3390/genes9030137 29498629 PMC5867858

[4] Wada J, Kanwar YS. Identification and characterization of galectin-9, a novel beta-galactoside-binding mammalian lectin. J Biol Chem. 1997; 272:6078–86. 10.1074/jbc.272.9.6078 9038233

[5] Chou FC, Chen HY, Kuo CC, Sytwu HK. Role of Galectins in Tumors and in Clinical Immunotherapy. Int J Mol Sci. 2018; 19:E430. 10.3390/ijms19020430 29389859 PMC5855652

[6] Wolf Y, Anderson AC, Kuchroo VK. TIM3 comes of age as an inhibitory receptor. Nat Rev Immunol. 2020; 20:173–85. 10.1038/s41577-019-0224-6 31676858 PMC7327798

[7] Zhu C, Anderson AC, Schubart A, Xiong H, Imitola J, Khoury SJ, Zheng XX, Strom TB, Kuchroo VK. The Tim-3 ligand galectin-9 negatively regulates T helper type 1 immunity. Nat Immunol. 2005; 6:1245–52. 10.1038/ni1271 16286920

[8] Wu C, Thalhamer T, Franca RF, Xiao S, Wang C, Hotta C, Zhu C, Hirashima M, Anderson AC, Kuchroo VK. Galectin-9-CD44 interaction enhances stability and function of adaptive regulatory T cells. Immunity. 2014; 41:270–82. 10.1016/j.immuni.2014.06.011 25065622 PMC4219323

[9] Daley D, Mani VR, Mohan N, Akkad N, Ochi A, Heindel DW, Lee KB, Zambirinis CP, Pandian GS, Savadkar S, Torres-Hernandez A, Nayak S, Wang D, et al. Dectin 1 activation on macrophages by galectin 9 promotes pancreatic carcinoma and peritumoral immune tolerance. Nat Med. 2017; 23:556–67. 10.1038/nm.4314 28394331 PMC5419876

[10] Sun Q, Zhang Y, Liu M, Ye Z, Yu X, Xu X, Qin Y. Prognostic and diagnostic significance of galectins in pancreatic cancer: a systematic review and meta-analysis. Cancer Cell Int. 2019; 19:309. 10.1186/s12935-019-1025-5 31832021 PMC6873495

[11] Irie A, Yamauchi A, Kontani K, Kihara M, Liu D, Shirato Y, Seki M, Nishi N, Nakamura T, Yokomise H, Hirashima M. Galectin-9 as a prognostic factor with antimetastatic potential in breast cancer. Clin Cancer Res. 2005; 11:2962–8. 10.1158/1078-0432.CCR-04-0861 15837748

[12] Schulz H, Kuhn C, Hofmann S, Mayr D, Mahner S, Jeschke U, Schmoeckel E. Overall Survival of Ovarian Cancer Patients Is Determined by Expression of Galectins-8 and -9. Int J Mol Sci. 2018; 19:E323. 10.3390/ijms19010323 29361803 PMC5796266

[13] Ohue Y, Kurose K, Nozawa R, Isobe M, Nishio Y, Tanaka T, Doki Y, Hori T, Fukuoka J, Oka M, Nakayama E. Survival of Lung Adenocarcinoma Patients Predicted from Expression of PD-L1, Galectin-9, and XAGE1 (GAGED2a) on Tumor Cells and Tumor-Infiltrating T Cells. Cancer Immunol Res. 2016; 4:1049–60. 10.1158/2326-6066.CIR-15-0266 27799141

[14] Li H, Wu K, Tao K, Chen L, Zheng Q, Lu X, Liu J, Shi L, Liu C, Wang G, Zou W. Tim-3/galectin-9 signaling pathway mediates T-cell dysfunction and predicts poor prognosis in patients with hepatitis B virus-associated hepatocellular carcinoma. Hepatology. 2012; 56:1342–51. 10.1002/hep.25777 22505239

[15] Sideras K, Biermann K, Verheij J, Takkenberg BR, Mancham S, Hansen BE, Schutz HM, de Man RA, Sprengers D, Buschow SI, Verseput MC, Boor PP, Pan Q, et al. PD-L1, Galectin-9 and CD8^+^ tumor-infiltrating lymphocytes are associated with survival in hepatocellular carcinoma. OncoImmunology. 2017; 6:e1273309. 10.1080/2162402X.2016.1273309 28344887 PMC5353918

[16] Sideras K, de Man RA, Harrington SM, Polak WG, Zhou G, Schutz HM, Pedroza-Gonzalez A, Biermann K, Mancham S, Hansen BE, Bart Takkenberg R, van Vuuren AJ, Pan Q, et al. Circulating levels of PD-L1 and Galectin-9 are associated with patient survival in surgically treated Hepatocellular Carcinoma independent of their intra-tumoral expression levels. Sci Rep. 2019; 9:10677. 10.1038/s41598-019-47235-z 31337865 PMC6650499

[17] Zhang ZY, Dong JH, Chen YW, Wang XQ, Li CH, Wang J, Wang GQ, Li HL, Wang XD. Galectin-9 acts as a prognostic factor with antimetastatic potential in hepatocellular carcinoma. Asian Pac J Cancer Prev. 2012; 13:2503–9. 10.7314/APJCP.2012.13.6.2503 22938412

[18] Jiao D, Li Y, Yang F, Han D, Wu J, Shi S, Tian F, Guo Z, Xi W, Li G, Zhao A, Yang AG, Qin W, et al. Expression of Prostate-Specific Membrane Antigen in Tumor-Associated Vasculature Predicts Poor Prognosis in Hepatocellular Carcinoma. Clin Transl Gastroenterol. 2019; 10:1–7. 10.14309/ctg.0000000000000041 31116141 PMC6602770

[19] Shi SJ, Wang LJ, Han DH, Wu JH, Jiao D, Zhang KL, Chen JW, Li Y, Yang F, Zhang JL, Zheng GX, Yang AG, Zhao AZ, et al. Therapeutic effects of human monoclonal PSMA antibody-mediated TRIM24 siRNA delivery in PSMA-positive castration-resistant prostate cancer. Theranostics. 2019; 9:1247–63. 10.7150/thno.29884 30867828 PMC6401511

[20] Yang S, Wang J, Chen F, Liu G, Weng Z, Chen J. Elevated Galectin-9 Suppresses Th1 Effector Function and Induces Apoptosis of Activated CD4^+^ T Cells in Osteoarthritis. Inflammation. 2017; 40:1062–71. 10.1007/s10753-017-0549-x 28393295

[21] Dardalhon V, Anderson AC, Karman J, Apetoh L, Chandwaskar R, Lee DH, Cornejo M, Nishi N, Yamauchi A, Quintana FJ, Sobel RA, Hirashima M, Kuchroo VK. Tim-3/galectin-9 pathway: regulation of Th1 immunity through promotion of CD11b+Ly-6G+ myeloid cells. J Immunol. 2010; 185:1383–92. 10.4049/jimmunol.0903275 20574007 PMC2925247

[22] Yang R, Sun L, Li CF, Wang YH, Yao J, Li H, Yan M, Chang WC, Hsu JM, Cha JH, Hsu JL, Chou CW, Sun X, et al. Galectin-9 interacts with PD-1 and TIM-3 to regulate T cell death and is a target for cancer immunotherapy. Nat Commun. 2021; 12:832. 10.1038/s41467-021-21099-2 33547304 PMC7864927

[23] Komita H, Koido S, Hayashi K, Kan S, Ito M, Kamata Y, Suzuki M, Homma S. Expression of immune checkpoint molecules of T cell immunoglobulin and mucin protein 3/galectin-9 for NK cell suppression in human gastrointestinal stromal tumors. Oncol Rep. 2015; 34:2099–105. 10.3892/or.2015.4149 26239720

[24] Wang Y, Sun J, Ma C, Gao W, Song B, Xue H, Chen W, Chen X, Zhang Y, Shao Q, Wang Q, Zhao L, Liu J, et al. Reduced Expression of Galectin-9 Contributes to a Poor Outcome in Colon Cancer by Inhibiting NK Cell Chemotaxis Partially through the Rho/ROCK1 Signaling Pathway. PLoS One. 2016; 11:e0152599. 10.1371/journal.pone.0152599 27028892 PMC4814049

[25] Yuan F, Ming H, Wang Y, Yang Y, Yi L, Li T, Ma H, Tong L, Zhang L, Liu P, Li J, Lin Y, Yu S, et al. Molecular and clinical characterization of Galectin-9 in glioma through 1,027 samples. J Cell Physiol. 2020; 235:4326–34. 10.1002/jcp.29309 31609000 PMC7028024

[26] Klibi J, Niki T, Riedel A, Pioche-Durieu C, Souquere S, Rubinstein E, Le Moulec S, Guigay J, Hirashima M, Guemira F, Adhikary D, Mautner J, Busson P. Blood diffusion and Th1-suppressive effects of galectin-9-containing exosomes released by Epstein-Barr virus-infected nasopharyngeal carcinoma cells. Blood. 2009; 113:1957–66. 10.1182/blood-2008-02-142596 19005181

[27] Chen P, Zhang L, Zhang W, Sun C, Wu C, He Y, Zhou C. Galectin-9-based immune risk score model helps to predict relapse in stage I-III small cell lung cancer. J Immunother Cancer. 2020; 8:e001391. 10.1136/jitc-2020-001391 33082168 PMC7577067

[28] Qi Y, Chang Y, Wang Z, Chen L, Kong Y, Zhang P, Liu Z, Zhou Q, Chen Y, Wang J, Bai Q, Xia Y, Liu L, et al. Tumor-associated macrophages expressing galectin-9 identify immunoevasive subtype muscle-invasive bladder cancer with poor prognosis but favorable adjuvant chemotherapeutic response. Cancer Immunol Immunother. 2019; 68:2067–80. 10.1007/s00262-019-02429-2 31720813 PMC11028176

[29] Li X, Chen Y, Liu X, Zhang J, He X, Teng G, Yu D. Tim3/Gal9 interactions between T cells and monocytes result in an immunosuppressive feedback loop that inhibits Th1 responses in osteosarcoma patients. Int Immunopharmacol. 2017; 44:153–9. 10.1016/j.intimp.2017.01.006 28103502

[30] Severson JJ, Serracino HS, Mateescu V, Raeburn CD, McIntyre RC Jr, Sams SB, Haugen BR, French JD. PD-1+Tim-3+ CD8+ T Lymphocytes Display Varied Degrees of Functional Exhaustion in Patients with Regionally Metastatic Differentiated Thyroid Cancer. Cancer Immunol Res. 2015; 3:620–30. 10.1158/2326-6066.CIR-14-0201 25701326 PMC4457654

[31] Okoye I, Xu L, Motamedi M, Parashar P, Walker JW, Elahi S. Galectin-9 expression defines exhausted T cells and impaired cytotoxic NK cells in patients with virus-associated solid tumors. J Immunother Cancer. 2020; 8:e001849. 10.1136/jitc-2020-001849 33310773 PMC7735134

[32] Wiersma VR, de Bruyn M, Helfrich W, Bremer E. Therapeutic potential of Galectin-9 in human disease. Med Res Rev. 2013 (Suppl 1); 33:E102–26. 10.1002/med.20249 21793015

[33] Manero-Rupérez N, Martínez-Bosch N, Barranco LE, Visa L, Navarro P. The Galectin Family as Molecular Targets: Hopes for Defeating Pancreatic Cancer. Cells. 2020; 9:E689. 10.3390/cells9030689 32168866 PMC7140611

[34] Selnø AT, Schlichtner S, Yasinska IM, Sakhnevych SS, Fiedler W, Wellbrock J, Klenova E, Pavlova L, Gibbs BF, Degen M, Schnyder I, Aliu N, Berger SM, et al. Transforming growth factor beta type 1 (TGF-β) and hypoxia-inducible factor 1 (HIF-1) transcription complex as master regulators of the immunosuppressive protein galectin-9 expression in human cancer and embryonic cells. Aging (Albany NY). 2020; 12:23478–96. 10.18632/aging.202343 33295886 PMC7762483

[35] Chen S, Pu J, Bai J, Yin Y, Wu K, Wang J, Shuai X, Gao J, Tao K, Wang G, Li H. EZH2 promotes hepatocellular carcinoma progression through modulating miR-22/galectin-9 axis. J Exp Clin Cancer Res. 2018; 37:3. 10.1186/s13046-017-0670-6 29316949 PMC5761110

