# Supplementary material for: Galectin-9 expression predicts poor prognosis in hepatitis B virus-associated hepatocellular carcinoma
Source: Aging (Albany NY). 2022 Feb 24;14(4):1879–90. doi: 10.18632/aging.203909 (PMC8908941; doi:10.18632/aging.203909)
Supplement: Supplementary Methods [file aging-14-203909-s001.pdf]

SUPPLEMENTARY METHODS

Immunohistochemistry (IHC) staining

Paraffin-embedded HCC tissue wax blocks were cut into 4 µm thick tissue pieces and placed on glass sides. Slides were then subjected to IHC staining to evaluate Gal-9 expression. Briefly, slides were deparaffinized in xylene, and rehydrated through gradient ethanol series (100%, 95%, 90%, 80%, 70%); then, antigen retrieval was performed under high temperature and pressure in sodium citrate buffer (0.1mol/L citric acid /0.1mol/L sodium citrate, pH 6.0) for 90 seconds. After endogenous peroxidase was inactivated using blocking solution (SP KIT-A3, MXB Biotechnologies), slides were washed by phosphate buffered saline (PBS) for 3 times. Then, slides were blocked with nonimmune goat serum (SP KIT-B3, MXB Biotechnologies) for 20 minutes at room temperature and incubated with anti-Gal-9 antibody (#54330, Cell Signaling Technology) in a humidified chamber overnight at 4° C. After being washed 3 times with PBS, slides were then incubated with horseradish peroxidase (HRP)-labeled goat anti-mouse/rabbit IgG (KIT-5030, MXB Biotechnologies) at room temperature for 30 minutes, followed by 3 times washes with PBS. Visualization reaction was conducted by the application of Diaminobenzidine (DAB, DAB-4033, MXB Biotechnologies) and then stopped for 3 minutes in tap water. Slides were then counterstained with hematoxylin for 5 minutes, raised in water, dehydrated in ascending concentration of ethanol (70%, 80%, 90%, 95%, 100%), followed by clearance with xylene, and cover slip permanently for light microscopy observation. Positive control and negative control were set in each group in order to avoid false positive or false negative results. Slides were digitally scanned using Nano Zoomer SQ workstation at the ×40 setting (HAMAMATSU, Inc).

Immunohistochemistry evaluation

The staining intensity (0=negative, 1=weak, 2=moderate, and 3=strong) and percentage of positive cells (0=0%; 1=1-25%; 2=26-50%; 3=51-75%; 4=76-100% stained cells) were evaluated and multiplied to obtain an immunoreactivity score. Samples with score≥6 were grouped as strong Gal-9 expression, samples with 6>score≥3 were grouped as moderate Gal-9 expression, samples with 3>score>0 were grouped as weak Gal-9 expression, and samples with score=0 were grouped as negative Gal-9 expression. For statistical analysis, samples with strong, moderate or weak Gal-9 expression were all grouped as "positive Gal-9 expression" (score>0), and samples with no Gal-9 expression were grouped as "negative Gal-9 expression" (score=0). For survival analysis, patients were also divided into two groups according to the same principle, that is Gal-9 positive group (score>0) and Gal-9 negative group (score=0).

Immunofluorescent staining

Paraffin-embedded HCC tissue slides were prepared as above. Slides were also deparaffinized, rehydrated, antigen retrieved, and blocked endogenous peroxidase and nonspecific protein binding as above. Then slides were incubated with corresponding primary antibodies in a humidified chamber overnight at 4° C. Slides were then washed 3 times with PBS before they were incubated with Cy3/FITC-conjugated goat anti-rabbit IgG secondary antibody (GB23303, Wuhan Servicebio Technology, 1:500) for 1 hour at 4° C in darkness. Next, slides were stained with 4´,6-diamidino-2-phenylindole (DAPI) to visualize the nuclei. Finally, slides were sealed by anti-fluorescence quenching seal tablet (G1401, Wuhan Servicebio Technology) before they were observed and imaged under a laser scanning confocal microscope (NIKON ECLIPSE C1, Nikon Corporation, Japan) and an imaging system (NIKON DS-U3, Nikon Corporation, Japan). Antibodies used for immunofluorescent staining were: Gal-9 antibody (#54330, Cell Signaling Technology; 1:400), CD68 antibody (GB13063-1, Wuhan Servicebio Technology, 1:2000), Glypican 3 (GPC3) antibody (ab95363, Abcam, 1:100), CD163 antibody (Ab156769, Abcam, 1:150) and CD206 antibody (GB13438, Wuhan Servicebio Technology, 1:2000).

Evaluation of immunofluorescent staining

Pearson's correlation coefficient (PCC) was acquired in region of interest (ROI). The number "1" represents perfect correlation (protein B is necessary where protein A is present); the number "-1" represents complete exclusion (protein B is absent where protein A is present), and zero represents random relationship (protein A and protein B are randomly distributed, no correlation). Scatterplot analysis was also displayed in a 2D intensity histogram.
